# Associations Between Vitamin D Deficiency and Sarcopenia in South Korean Adults: Based on the 2022 Korea National Health and Nutrition Examination Survey

**DOI:** 10.3390/nu17203292

**Published:** 2025-10-20

**Authors:** Sunhye Shin, Mi Joung Kim

**Affiliations:** Department of Food and Nutrition, Seoul Women’s University, Seoul 01797, Republic of Korea; sshin@swu.ac.kr

**Keywords:** vitamin D, sarcopenia, low muscle mass, low muscle strength, 25-hydroxyvitamin D, KNHANES

## Abstract

**Background/Objectives**: Although vitamin D has been associated with sarcopenia in older adults, evidence across age groups remains limited. This study evaluated the relationship between vitamin D deficiency (VDD) and muscle health in Korean adults aged ≥19 years. **Methods**: Data utilized in this study were obtained from the 2022 Korea National Health and Nutrition Examination Survey IX-1. Serum 25-hydroxyvitamin D [25(OH)D] levels were analyzed in relation to appendicular skeletal muscle mass (ASM), grip strength, and sarcopenia using multivariable regression models. **Results**: Among 3,920 participants, 46.5% had VDD, with the highest prevalence observed in younger adults. After adjusting for age, body mass index, energy intake, and other confounding factors, serum 25(OH)D levels showed a positive association with ASM in middle-aged men (β = 0.005; *p* = 0.007) and with maximal handgrip strength in young men (β = 0.097; *p* = 0.048). Among older men, those with VDD had significantly higher odds of low muscle mass (OR = 1.82; 95% CI: 1.10–3.02) and sarcopenia (OR = 2.30; 95% CI: 1.03–5.16) than those without VDD, after adjusting for potential confounders. No significant associations were observed in women. **Conclusions**: These results suggest that maintaining adequate vitamin D levels may benefit muscle health in men. Further prospective or interventional studies are needed to more accurately assess the effects of vitamin D on muscle health.

## 1. Introduction

Sarcopenia is a progressive and age-associated degenerative condition characterized by the loss of skeletal muscle mass and strength [1]. In older adults, sarcopenia leads to adverse outcomes, including falls, diminished quality of life, and increased mortality, and is associated with chronic conditions, such as cardiovascular disease and diabetes [1,2,3,4]. Sarcopenia has gained increasing clinical importance among the aging population. The concept of “probable sarcopenia”—indicating decreased muscle strength—has recently been introduced to screen high-risk groups [5].

Sarcopenia is a natural consequence of aging and is influenced by a combination of diverse factors, including health-related lifestyle factors, chronic diseases, and nutritional status [6,7]. Among these factors, vitamin D is an essential nutrient for maintaining skeletal muscle function. Acting via the vitamin D receptor (VDR), it promotes muscle protein synthesis and supports muscle cell growth and differentiation [8]. In muscle tissues, vitamin D regulates mitochondrial DNA stability and energy metabolism, enhancing adenosine triphosphate (ATP) production and reducing oxidative stress to preserve function [9]. It also activates anti-inflammatory cells (including M2 macrophages) and inhibits proinflammatory cytokines, such as interferon-γ, interleukin-1, interleukin-6, and tumor necrosis factor-α, preventing muscle cell damage [9]. Consequently, vitamin D deficiency (VDD) is associated with muscle weakness, functional decline, and increased risk of developing sarcopenia [7,8,9]. Conversely, vitamin D supplementation supports muscle cell regeneration and functional recovery by reducing reactive oxygen species (ROS), enhancing antioxidant capacity, and promoting mitochondrial ATP production [10].

Particularly, South Korea has a high prevalence of VDD, attributable to irregular dietary habits and increased indoor activity. Approximately 50% of Korean adults have serum 25-hydroxyvitamin D_3_ [25(OH)D] concentrations <20 ng/mL, with deficiency rates higher in younger adults than in older adults [11]. Moreover, the average dietary vitamin D intake in Koreans is <50% of the recommended level [12].

Although VDD has been linked to osteoporosis and other chronic disease incidence [13,14], few large-scale, population-based studies have investigated the association between vitamin D status and muscle strength or sarcopenia, particularly with age stratification. Notably, as sarcopenia risk begins to increase in early adulthood, studies should include both younger and older adults. Such studies should also account for potential confounding factors affecting muscle health, including protein intake and resistance exercise.

Therefore, this study analyzed data from the 9th Korea National Health and Nutrition Examination Survey (KNHANES IX-1, 2022)—including dietary intake, serum 25(OH)D levels, and handgrip strength measurements—to test the hypothesis that VDD is associated with low muscle strength in adults aged ≥19 years.

## 2. Materials and Methods

### 2.1. Data Source and Participants

**Figure 1:**
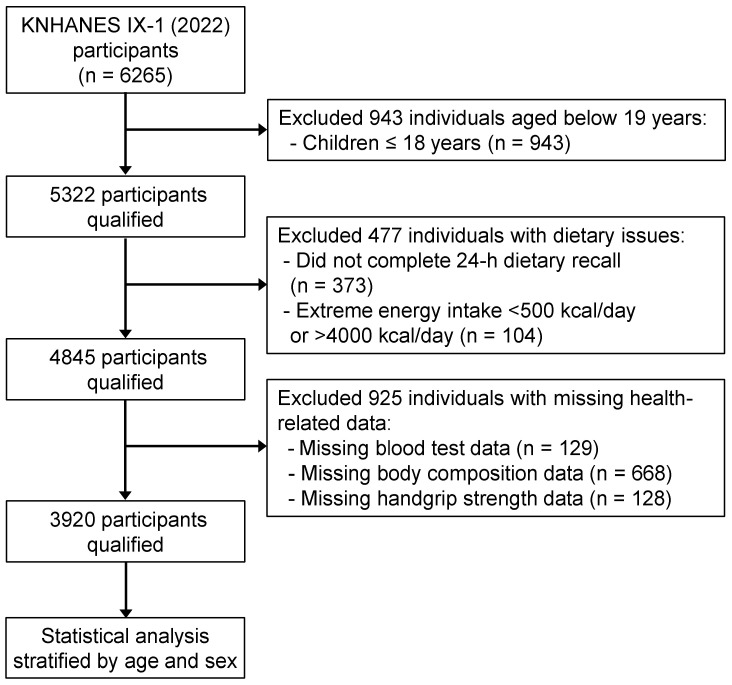
Selection process of study participants. Flow diagram showing the number of included and excluded participants and the data for analysis. KNHANES: Korea National Health and Nutrition Examination Survey; *n* = unweighted numbers.

The KNHANES is a cross-sectional survey designed to assess the health and nutritional status of a nationally representative sample of Koreans aged ≥1 year [15,16]. For this study, we analyzed data from the KNHANES IX-1 (2022). Among 6265 participants of KNHANES IX-1, children aged <18 years (*n* = 943), who did not complete a 24 h dietary recall (*n* = 373), those with extreme energy intake (<500 kcal/d or >4000 kcal/d; *n* = 104), those with missing blood test results data (*n* = 129), those without body composition data (*n* = 668), and those without handgrip strength information (*n* = 128) were subsequently excluded. A final sample of 3920 participants was stratified based on age and sex to adjust for possible biological differences: younger men aged 19–39 years (*n* = 447), younger women aged 19–39 years (*n* = 549), middle-aged men (*n* = 731), middle-aged women (*n* = 1057), older men aged ≥65 years (*n* = 547), and older women aged ≥65 years (*n* = 559) (Figure 1).

This study was conducted in accordance with the Declaration of Helsinki. The Institutional Review Board of the Korea Centers for Disease Control and Prevention approved the KNHANES data collection procedures (IRB No. 2018-01-03-4C-A). All participants provided written informed consent. As this study involved secondary analyses of KNHANES data, ethical review and approval were waived by the Institutional Review Board of Seoul Women’s University (No. SWU IRB-2025A-35).

### 2.2. Vitamin D Status, Body Composition, and Handgrip Strength Measures

For vitamin D status, serum 25(OH)D level was measured using liquid chromatography–mass spectrometry. Serum 25(OH)D < 20 ng/mL was classified as VDD according to the established consensus [17,18,19].

Body composition was estimated using bioelectrical impedance analysis (BIA, Inbody 970, InBody Co., Ltd, Seoul, Republic of Korea). Appendicular skeletal muscle mass (ASM) was estimated by summing the muscle mass of both upper and lower limbs bilaterally. ASM index was calculated as appendicular skeletal muscle mass divided by height squared.

Handgrip strength was assessed using the digital grip strength dynamometer (T.K.K.5401, Takei Scientific Instruments Co., Ltd, Tokyo, Japan) with participants in an upright position and their elbow fully extended. The highest value from two trials performed on the dominant hand was recorded in kilograms (kg). Participants determined their own eligibility for the test based on predefined criteria, including absence of acute injury, recent surgery, amputation, or paralysis.

Based on the 2019 consensus of the Asian Working Group for Sarcopenia [5], low muscle mass was defined as an ASM index < 7.0 kg/m^2^ for men and <5.7 kg/m^2^ for women, and low muscle strength as handgrip strength < 28 kg for men and <18 kg for women. Sarcopenia was characterized by loss of muscle mass and low muscle strength.

### 2.3. Covariate Measures

Information on demographic, socioeconomic, and lifestyle factors was collected during an in-person health interview using a structured questionnaire. Household income was classified into quartiles: low, middle-low, middle-high, and high. Participants who reported drinking alcohol more than once per month in the preceding year before the interview were considered current alcohol consumers. Current smokers were defined as individuals who had smoked >100 cigarettes in their lifetime and continued to smoke during the survey. Regular resistance exercise was defined as performing strength training activities (e.g., push-ups, sit-ups, deadlifts, and chin-ups) more than two times per week.

Body weight and height were measured during the health examination. Body mass index (BMI) was defined as weight (kg) divided by the square of height in meters (kg/m^2^).

Dietary intake was assessed using a single 24 h dietary recall. The calculation of daily intake levels for total energy, specific foods, and individual nutrients was conducted based on the recipe information and food composition database provided by the Korean Rural Development Administration [20], as utilized in the KNHANES.

### 2.4. Statistical Analyses

All statistical analyses were conducted using IBM SPSS Statistics for Windows, version 29 (IBM Corp., Armonk, NY, USA). After adjusting for survey design effects—including strata, clusters, and weights—complex samples general linear regression and complex samples logistic regression analyses were conducted to estimate β-coefficients for continuous variables and odds ratios (ORs) for categorical variables, respectively. A two-sided *p* < 0.05 was considered statistically significant, with exact *p*-values presented to enhance interpretation.

General characteristics were described using vitamin D status as means or percentages ± standard errors (SEs). General characteristic differences between normal and VDD participants were determined using the Rao–Scott chi-square tests and *t*-tests for categorical and continuous variables, respectively.

The association between serum vitamin D level and ASM or handgrip strength was analyzed using multivariate linear regression models and that between vitamin D status and risks of low muscle strength, low muscle mass, or sarcopenia was assessed using multivariate logistic regression models with the normal group as the reference. The unadjusted model estimated crude OR and 95% confidence intervals (CIs). Model 1 was adjusted for age, BMI, and total energy intake, while Model 2 was further adjusted for household income, current alcohol consumption, current smoking status, and participation in regular resistance exercise. Model 3 included all covariates from Model 2, with the addition of energy from protein, a recognized preventive factor against low muscle mass [21]. Duration of sun exposure, seasonal variation, ultraviolet index, medical history (e.g., type 2 diabetes, osteoarthritis, and chronic kidney disease), use of vitamin D supplements and steroid medications, and levels of parathyroid hormone (PTH), calcium, and endogenous hormones (e.g., testosterone and estradiol) are associated with vitamin D status and muscle health [12,14,18,22]. However, these variables were not included in the KNHANES IX-1 dataset and thus could not be accounted for in the regression analyses.

## 3. Results

Approximately half of Korean adults (46.5%) had serum 25(OH)D level < 20 ng/mL. VDD prevalence by age and sex was 63.2% in younger men (19–39 years), 60.7% in younger women (19–39 years), 46.1% in middle-aged men (40–64 years), 36.8% in middle-aged women (40–64 years), 38.4% in older men (≥65 years), and 27.6% in older women (≥65 years), respectively. The average dietary vitamin D intake of Korean adults was 3.0 μg/d, far below the adequate intake (AI) of vitamin D (10 μg/d for young and middle-aged adults and 15 μg/d for older adults), and <5% met AI for vitamin D via diets (≥AI; Supplementary Table S1).

Low muscle mass, low muscle strength, and sarcopenia (low muscle mass plus low muscle strength) prevalence among Korean adults were 16.7%, 4.8%, and 2.5%, respectively. Older women had the highest low muscle mass, low muscle strength, and sarcopenia prevalence, followed by older men, younger women, middle-aged women, younger men, and middle-aged men (Supplementary Table S1).

Among young adults aged 19–39 years, individuals with VDD were on an average younger than those without the deficiency. Younger men with VDD were less likely to perform regular resistance exercise and had lower maximal handgrip strength. Younger women with VDD were more likely to be current smokers. Middle-aged men with VDD were more likely to be current smokers, and middle-aged women with VDD had higher BMI. Older men with VDD had lower maximal handgrip strength and higher risks of low muscle mass, low muscle strength, and sarcopenia. Older women with VDD had higher BMI. No age- or sex-group differences were observed in daily mean dietary vitamin D intake between participants with and without VDD (Supplementary Tables S2–S4).

Serum 25(OH)D level was positively correlated with ASM index in middle-aged men (40–64 years; β = 0.005; 95% CI, 0.001–0.009; *p* = 0.007). Blood 25(OH)D level was positively correlated with maximal handgrip strength in younger men (19–39 years; β = 0.097; 95% CI, 0.001–0.194; *p* = 0.048) after adjusting for confounding variables, including age, BMI, energy intake, household income, alcohol, smoking, resistance exercise, and energy from protein. No correlation was observed in women, regardless of age (Table 1 and Table 2).

Although the serum 25(OH)D level, as a continuous variable, was not correlated with muscle mass and strength among older men, VDD was associated with a low muscle mass and sarcopenia prevalence among older men. After adjusting for potential confounders, older men with VDD had a 1.82 times higher risk of low muscle mass (OR, 1.82; 95% CI, 1.10–3.02) and a 2.30 times higher risk of sarcopenia (OR, 2.30; 95% CI, 1.03–5.16) than those without VDD (Table 3). When older adults were divided into three groups—normal, vitamin D insufficiency (serum 25(OH)D level 20–30 ng/mL), and VDD—the vitamin D insufficiency group was not associated with a higher risk of low muscle mass, low muscle strength, or sarcopenia (Supplementary Table S5).

## 4. Discussion

Sarcopenia is a common age-related condition that impairs daily function, increases fall risk, and raises mortality risk in older adults. Analysis of the KNHANES 2022–2023 data reported sarcopenia in approximately 11% of Koreans aged ≥ 65 years [23]. VDD is also widespread globally and particularly prevalent in Korea. A recent study found a median serum 25(OH)D concentration of 20.2 ng/mL among Korean adults, with 49.4% classified as VDD (serum 25(OH)D < 20 ng/mL) [24]. Vitamin D insufficiency (serum 25(OH)D < 30 ng/mL) was most prevalent among adults aged 20–40 years [24]. Similarly, our study showed that 46.5% of participants had VDD, with a higher prevalence in younger adults than in older adults. This trend likely reflects lifestyle changes, including increased time spent indoors, limited cutaneous vitamin D synthesis owing to air pollution and high-rise buildings, and dietary shifts that reduce vitamin D intake [11,24].

This study demonstrated a positive association between vitamin D status and muscle health across different age groups among Korean adults. Our hypothesis that VDD would be associated with lower muscle mass and strength was supported by findings in both young (19–39 years) and middle-aged (40–64 years) male participants. Serum 25(OH)D concentrations were positively correlated with handgrip strength in young men and with ASM in middle-aged men, after controlling for age, BMI, energy intake, and other confounders. Based on the β coefficients, a 10 ng/mL increase in serum 25(OH)D was associated with an approximate 0.05 kg/m^2^ increase in ASM index among middle-aged men (β = 0.005). In younger men, the same increment in 25(OH)D corresponded to an estimated 0.97 kg increase in maximal handgrip strength (β = 0.097). Notably, in older men, even after controlling for potential confounders, VDD was associated with a 1.82-fold and 2.3-fold higher risk of low muscle mass and sarcopenia, respectively. When translated into predicted probabilities, assuming a baseline prevalence of 10% for low muscle mass and sarcopenia among vitamin D–sufficient older men, VDD was estimated to increase the predicted probabilities of low muscle mass and sarcopenia to approximately 17–18% and 20%, respectively. These estimates suggest the potential impact of maintaining adequate vitamin D levels on muscle health in men.

The average dietary vitamin D intake in our study population was approximately 3.0 μg/d, roughly 20–30% of the Korean AI across all age groups. Men and women in the VDD group showed a nonsignificant trend toward lower dietary vitamin D intake than those without the deficiency. Similarly, the KNHANES 2022–2023 data reported low vitamin D intake in older adults and showed that older adults with sarcopenia consumed significantly less protein, calcium, magnesium, and other micronutrients than those without sarcopenia; particularly, men with sarcopenia had significantly lower vitamin D intake and reduced physical activity levels [23]. These findings suggest that sarcopenia is influenced by vitamin D intake, poor overall diet quality (e.g., low protein and micronutrient intake), and physical inactivity, likely contributing to the loss of muscle mass and function.

Ahn et al. [11], analyzing data from the KNHANES, reported that although the prevalence of VDD among Korean adults has slightly decreased recently, the proportion of individuals with vitamin D insufficiency (<30 ng/mL) has increased. They suggested that younger adults have the highest prevalence of deficiency due to urban lifestyles and limited sun exposure. Conversely, older adults have a relatively lower prevalence due to a higher proportion of rural residents and greater use of supplements. Consistent with these findings, our study also showed that younger participants had a higher prevalence of VDD than older adults, suggesting that lifestyle and behavioral factors influence vitamin D status across all age groups.

Similarly to our findings, many observational studies have reported a significant association between VDD and sarcopenia [25,26]. A case–control study among Chinese adults aged ≥ 60 years found that VDD was associated with a 7.75-fold increased sarcopenia risk, and serum 25(OH)D levels were positively correlated with muscle mass and function indices [25]. Similarly, a prospective study in postmenopausal women found that VDD was associated with significantly lower appendicular muscle strength, weaker handgrip strength, and reduced knee extension power [27]. Consistent with our results, several studies have also demonstrated that lower 25(OH)D levels are associated with reduced muscle mass, decreased muscle strength, and impaired physical performance [26,28,29].

Kim et al. [30] reported that, in a 4-year follow-up study of Korean adults, both younger and older adults with low vitamin D status had an increased risk of muscle loss, and this risk was reduced when vitamin D status was improved. In addition, Yu et al. [31] suggested that chronic vitamin D deficiency may increase the risk of age-related muscle loss. In other words, low vitamin D status at all ages may be associated with muscle loss [30] and age-related muscle wasting [31]. Therefore, although sarcopenia generally occurs in older adults aged over 60 years, maintaining adequate vitamin D levels from early adulthood may be effective in maintaining muscle health and preventing sarcopenia in older adults. Therefore, it is considered necessary to develop nutrition and practical education programs—applicable from early adulthood—such as those including guidance on adequate vitamin D intake and regular outdoor physical activity, to help maintain sufficient vitamin D status.

No significant relationship was observed between serum 25(OH)D levels and muscle mass or strength in women of any age group. After controlling for potential confounding variables—specifically, the higher smoking prevalence among younger women with VDD, and the significantly younger age and elevated BMI among middle-aged and older women with VDD—no statistically significant associations were identified between serum 25(OH)D concentrations and muscle mass, muscle strength, or the presence of sarcopenia across any female age group. Similarly, Sponchiado et al. [32] and Kim et al. [33] reported a positive association between vitamin D status and muscle health in men, but not in women aged > 50 years. They proposed that this pattern could be attributed to sex-related differences in hormone profiles, lifestyle factors, and body composition, all of which may influence vitamin D metabolism and muscle function. Some evidence also indicates that vitamin D status may be more directly related to muscle mass and strength in men, whereas in women it may play a more prominent role in bone health and bone mineral density [34]. This perspective may partially align with our observations.

Moreover, our results showed no significant differences in the risk of low muscle mass, low muscle strength, or sarcopenia due to VDD, even among older women with the highest prevalence of VDD. This may be partly attributed to the fact that, in our results, older women had the highest serum 25(OH)D concentrations across all subgroups. Ahn et al. [11] also found that older Korean women tend to have higher vitamin D levels and lower deficiency rates, which they attributed to greater health awareness and more frequent supplement use. These factors may have attenuated or obscured any potential associations between vitamin D status and sarcopenia in women within our analysis.

Vitamin D not only plays a central role in bone metabolism but is also believed to exert effects on skeletal muscle through the VDR expressed in muscle tissue [8,35]. Additionally, vitamin D may reduce the production of ROS and enhance antioxidant capacity, potentially promoting recovery from muscle damage [10,36]. It may also help mitigate the oxidative stress, mitochondrial dysfunction, and activation of muscle atrophy signaling pathways observed during VDD.

However, the results of studies on vitamin D supplementation for muscle health remain inconsistent. Some studies have reported that vitamin D supplementation improved muscle strength and mass in older adults with severe VDD [37]. Conversely, other studies have shown that vitamin D supplementation alone had no significant overall effect on appendicular muscle mass, grip strength, or physical performance in older adults [38,39]. Sha et al. [40]. observed that the effect of vitamin D supplementation was evident only in individuals with low serum 25(OH)D levels and that the association between serum 25(OH)D and the risk of sarcopenia was nonlinear (specifically, L-shaped), suggesting that vitamin D supplementation may be effective only when serum levels are below approximately 20 ng/mL. Furthermore, vitamin D supplementation has been reported to be more effective when combined with other nutrients or interventions, such as protein supplementation or resistance exercise, rather than when used alone [37]. Additionally, Mądra-Gackowska et al. [41,42] reported changes in various indicators, including nutritional and inflammatory markers (such as serum albumin, *C*-reactive protein, and inflammatory cytokines), antioxidant-related markers (such as oxidized LDL and glutathione peroxidase), and adipokines in malnourished older individuals with sarcopenia. These findings suggest that the loss of muscle mass or sarcopenia in older people is not only associated with simple VDD but also with various nutritional factors, such as protein intake, and with age-related physiological changes, including alterations in antioxidant systems and inflammatory responses. Therefore, when considering our results as a whole—including the positive associations observed between vitamin D levels and muscle strength in young men, as well as the high prevalence of VDD in this group—these findings collectively suggest the need for further research, including prospective studies, to determine the precise effects of vitamin D supplementation and the baseline serum 25(OH)D concentration required for effective intervention.

In this study, we observed a cross-sectional association between low serum 25(OH)D concentrations and impaired muscle function; however, individuals with reduced muscle strength or impaired physical performance may spend less time outdoors, which could potentially reduce cutaneous vitamin D synthesis and lower serum 25(OH)D concentrations. Aspell et al. [43] supported this possibility by suggesting that reduced physical activity capacity is associated with lower vitamin D levels and that reduced activity might contribute to the development of VDD rather than being solely a consequence of it. Similarly, Mendes et al. [44] and Granic et al. [45], in cross-sectional and prospective studies of older adults, proposed that lower physical ability could limit sunlight exposure and dietary intake, thereby negatively affecting vitamin D status. In addition, Zhang et al. [2] suggested that changes in physical function associated with chronic diseases might influence muscle health. Taken together, these findings suggest that the association observed in this study between serum 25(OH)D and muscle health—particularly in older men—might have been overestimated due to factors not fully accounted for in our analysis.

As this study utilized data from the KNHANES IX-1, a key strength lies in its use of a large, nationally representative sample collected through validated and standardized methods. However, this study had some limitations, as it was constrained by its cross-sectional design, which prevented us from determining whether low vitamin D status leads to muscle health decline. Moreover, the KNHANES IX-1 dataset did not include a number of variables that could influence both vitamin D status and muscle health, such as duration of sunlight exposure, seasonal variation, ultraviolet index, season of blood sampling, geographic region, resistance exercise frequency, vitamin D supplementation, chronic diseases (e.g., chronic kidney disease, osteoarthritis, diabetes), medications (e.g., steroids, statins), parathyroid hormone and calcium levels, or endogenous hormone concentrations (e.g., testosterone, estradiol). Consequently, it was difficult to clearly assess the association between vitamin D levels and muscle health.

Furthermore, the KNHANES IX-1 dataset did not include information on key factors that could affect the accuracy of ASM measurements obtained using BIA, such as measurement time, fasting status, recent exercise, and acute illness. With regard to grip strength, participants with a history of prior injury or surgery could self-exclude from the measurement, which may have resulted in the study findings being more representative of relatively healthier individuals. In addition, resistance exercise frequency was assessed using a self-reported method, without detailed information, such as exercise intensity or routines, which may have made it difficult to fully account for the effects of resistance exercise on muscle health.

Menopausal status—which may influence muscle health—was not controlled for in this study due to both the reduced sample size within each stratum after stratification based on age and sex, as well as the lack of significant associations between vitamin D levels and muscle health in women across all age groups.

Although we performed multiple comparisons across sex- and age-specific subgroups and multiple outcome variables, we did not apply formal corrections for multiple testing, such as the Bonferroni correction. This decision was based on the hypothesis-driven nature of our study and the fact that the KNHANES analytic guidelines do not mandate such corrections in prevalence estimation or association studies. Nonetheless, the increased risk of type I error due to multiple comparisons should be considered when interpreting the findings.

Finally, the use of a single 24 h dietary recall may not accurately capture habitual total dietary intake or total vitamin D intake. Therefore, additional prospective or interventional studies are warranted to address these limitations.

## 5. Conclusions

We analyzed data from the KNHANES IX-1 to investigate the association between vitamin D status and sarcopenia. We observed positive associations of serum 25(OH)D levels with handgrip strength in young men and with ASM in middle-aged men. Furthermore, older men with VDD showed a higher risk of low muscle mass and sarcopenia than those without VDD. These findings suggest that maintaining adequate vitamin D status could be beneficial for supporting muscle health in men and for potentially reducing the risk of sarcopenia in later life. Further prospective studies are needed to clarify the effects of vitamin D on muscle health and that interventional studies could help evaluate the effects, timing, and dosage of vitamin D supplementation on muscle-related outcomes.

## Figures and Tables

**Table 1:** Association between serum 25(OH)D level and appendicular skeletal muscle index of participants based on age and sex.

Serum 25(OH)D Level	Unadjusted		Model 1 *		Model 2 ^†^		Model 3 ^‡^	
	β (95% CI)	*p*-Value	β (95% CI)	*p*-Value	β (95% CI)	*p*-Value	β (95% CI)	*p*-Value
Younger adults								
Men (*n* ^§^ = 447)	0.009 (0.000, 0.018)	0.061	0.010 (0.004, 0.016)	0.002	0.006 (0.000, 0.013)	0.058	0.006 (0.000, 0.013)	0.060
Women (*n* ^§^ = 549)	−0.004 (−0.011, 0.003)	0.241	0.003 (−0.001, 0.007)	0.204	0.003 (−0.001, 0.007)	0.184	0.003 (−0.001, 0.007)	0.180
Middle-aged adults								
Men (*n* ^§^ = 731)	−0.001 (−0.008, 0.006)	0.801	0.005 (0.001, 0.009)	0.007	0.005 (0.001, 0.009)	0.007	0.005 (0.001, 0.009)	0.007
Women (*n* ^§^ = 1057)	−0.007 (−0.011, −0.003)	0.002	<0.001 (−0.004, 0.004)	0.981	<0.001 (−0.004, 0.003)	0.841	<0.001 (−0.004, 0.003)	0.862
Older adults								
Men (*n* ^§^ = 547)	0.005 (−0.002, 0.011)	0.141	0.003 (0.000, 0.007)	0.086	0.003 (0.000, 0.007)	0.059	0.003 (0.000, 0.007)	0.079
Women (*n* ^§^ = 589)	−0.009 (−0.014, −0.004)	0.001	−0.001 (−0.005, 0.002)	0.404	−0.002 (−0.005, 0.001)	0.284	−0.002 (−0.005, 0.001)	0.269

Appendicular skeletal muscle index was calculated by dividing the sum of appendicular muscle mass by the height squared. * Model 1: adjusted for age, body mass index, and total energy intake. ^†^ Model 2: adjusted for all variables in model 1, with household income, alcohol consumption, smoking, and resistance exercise further included as covariates. ^‡^ Model 3: adjusted for all variables in model 2, with energy from protein further included as a covariate. ^§^ Unweight *n*. CI, confidence interval.

**Table 2:** Association between serum 25(OH)D level and maximal handgrip strength of participants based on age and sex.

Serum 25(OH)D Level	Unadjusted		Model 1 *		Model 2 ^†^		Model 3 ^‡^	
	β (95% CI)	*p*-Value	β (95% CI)	*p*-Value	β (95% CI)	*p*-Value	β (95% CI)	*p*-Value
Younger adults								
Men (*n* ^§^ = 447)	0.168 (0.064, 0.272)	0.002	0.151 (0.056, 0.246)	0.002	0.099 (0.003, 0.194)	0.043	0.097 (0.001, 0.194)	0.048
Women (*n* ^§^ = 549)	−0.011 (−0.060, 0.038)	0.668	−0.022 (−0.068, 0.024)	0.351	−0.018 (−0.065, 0.028)	0.437	−0.015 (−0.063, 0.032)	0.519
Middle-aged adults								
Men (*n* ^§^ = 731)	−0.005 (−0.065, 0.055)	0.874	0.020 (−0.036, 0.076)	0.475	0.017 (−0.041, 0.074)	0.567	0.017 (−0.040, 0.074)	0.555
Women (*n* ^§^ = 1057)	−0.025 (−0.048, −0.001)	0.041	0.004 (−0.022, 0.029)	0.784	−0.002 (−0.027, 0.024)	0.906	−0.002 (−0.028, 0.023)	0.853
Older adults								
Men (*n* ^§^ = 547)	0.048 (−0.006, 0.102)	0.081	0.029 (−0.020, 0.078)	0.241	0.033 (−0.015, 0.080)	0.179	0.028 (−0.020, 0.076)	0.251
Women (*n* ^§^ = 589)	0.017 (−0.016, 0.049)	0.322	0.026 (−0.003, 0.056)	0.083	0.020 (−0.009, 0.048)	0.171	0.018 (−0.010, 0.047)	0.202

* Model 1: adjusted for age, body mass index, and total energy intake. ^†^ Model 2: adjusted for all variables in model 1, with household income, alcohol consumption, smoking, and resistance exercise further included as covariates. ^‡^ Model 3: adjusted for all variables in model 2, with energy from protein further included as a covariate. ^§^ Unweight *n*. CI, confidence interval.

**Table 3:** Association between vitamin D deficiency and the risk of low muscle mass, low muscle strength, and sarcopenia among older adults.

Serum 25(OH)D Level	Unadjusted		Model 1 *		Model 2 ^†^		Model 3 ^‡^	
	OR	95% CI	OR	95% CI	OR	95% CI	OR	95% CI
Low muscle mass								
Men								
Normal (*n* ^§^ = 355)	1 (ref)		1 (ref)		1 (ref)		1 (ref)	
Vitamin D deficiency (*n* ^§^ = 192)	1.56	1.06, 2.28	1.77	1.08, 2.90	1.50	1.07, 3.02	1.82	1.10, 3.02
Women								
Normal (*n* ^§^ = 430)	1 (ref)		1 (ref)		1 (ref)		1 (ref)	
Vitamin D deficiency (*n* ^§^ = 159)	0.66	0.42, 1.04	0.80	0.45, 1.43	0.72	0.40, 1.27	0.71	0.40, 1.28
Low muscle strength								
Men								
Normal (*n* ^§^ = 355)	1 (ref)		1 (ref)		1 (ref)		1 (ref)	
Vitamin D deficiency (*n* ^§^ = 192)	2.05	1.15, 3.65	1.89	1.04, 3.46	1.84	1.01, 3.37	1.80	0.99, 3.26
Women								
Normal (*n* ^§^ = 430)	1 (ref)		1 (ref)		1 (ref)		1 (ref)	
Vitamin D deficiency (*n* ^§^ = 159)	1.53	0.89, 2.64	1.62	0.91, 2.88	1.39	0.75, 2.58	1.42	0.77, 2.62
Sarcopenia								
Men								
Normal (*n* ^§^ = 355)	1 (ref)		1 (ref)		1 (ref)		1 (ref)	
Vitamin D deficiency (*n* ^§^ = 192)	2.54	1.23, 5.25	2.58	1.18, 5.63	2.35	1.03, 5.33	2.30	1.03, 5.16
Women								
Normal (*n* ^§^ = 430)	1 (ref)		1 (ref)		1 (ref)		1 (ref)	
Vitamin D deficiency (*n* ^§^ = 159)	1.10	0.54, 2.24	1.21	0.54, 2.69	0.93	0.40, 2.13	0.94	0.41, 2.16

Vitamin D deficiency was defined as serum 25(OH)D < 20 ng/mL. Low muscle mass was defined as appendicular skeletal muscle index (appendicular skeletal muscle mass/height^2^) < 7.0 kg/m^2^ for men and <5.7 kg/m^2^ for women. * Model 1: adjusted for age, body mass index, and total energy intake. ^†^ Model 2: adjusted for all variables in model 1, with household income, alcohol consumption, smoking, and resistance exercise further included as covariates. ^‡^ Model 3: adjusted for all variables in model 2, with energy from protein further included as a covariate. ^§^ Unweight *n*. CI, confidence interval.

## Data Availability

The data presented in this study are available from the KNHANES database at https://knhanes.kdca.go.kr (accessed on 20 July 2025). Access to the data requires approval from the Korea Disease Control and Prevention Agency.

## Supplementary Materials

The following supporting information can be downloaded at: https://www.mdpi.com/article/10.3390/nu17203292/s1, Table S1: Prevalence of vitamin D deficiency and muscle-related disorders of participants by age and sex; Table S2: General characteristics of participants aged 19–39 years by vitamin D status; Table S3: General characteristics of participants aged 40–64 years by vitamin D status; Table S4: General characteristics of participants aged ≥ 65 years by vitamin D status; Table S5. Association between vitamin D status and the risk of low muscle mass, low muscle strength, and sarcopenia among older adults.

## Abbreviations

The following abbreviations are used in this manuscript:

VDR	vitamin D receptor
ATP	adenosine triphosphate
AI	adequate intake
BIA	bioelectrical impedance analysis
VDD	vitamin D deficiency
ROS	reactive oxygen species
25(OH)D	25-hydroxyvitamin D_3_
KNHANES	Korea National Health and Nutrition Examination Survey
PTH	parathyroid hormone
