# Supplementary material for: Associations Between Vitamin D Deficiency and Sarcopenia in South Korean Adults: Based on the 2022 Korea National Health and Nutrition Examination Survey
Source: Nutrients. 2025 Oct 20;17(20):3292. doi: 10.3390/nu17203292 (PMC12567185; doi:10.3390/nu17203292)
Supplement: Supplementary file 1 [file nutrients-17-03292-s001.zip › nutrients-3911872-supplementary.pdf]

Supplementary tables

Table S1. Prevalence of vitamin D deficiency and muscle-related disorders of participants based on age and sex

Characteristics	**Younger men (n = 447)**	**Younger women (n = 549)**	**Middle-aged men (n = 731)**	**Middle-aged women (n = 1057)**	**Older men (n = 547)**	**Older women (n = 589)**
Vitamin D deficiency*	276 (63.2)	326 (60.7)	322 (46.1)	390 (36.8)	192 (38.4)	159 (27.6)
Vitamin D insufficiency*	137 (28.9)	163 (29.1)	271 (34.9)	342 (32.4)	213 (37.3)	173 (28.6)
Adequate vitamin D intake†	22 (5.2)	18 (3.7)	52 (7.2)	36 (3.2)	11 (1.8)	7 (1.4)
Low muscle mass‡	41 (9.1)	147 (27.0)	51 (5.5)	165 (16.6)	159 (27.4)	172 (28.4)
Low muscle strength§	12 (2.7)	24 (3.9)	10 (1.1)	41 (3.2)	77 (12.9)	90 (13.7)
Sarcopenia‖	5 (1.2)	16 (2.4)	3 (0.3)	16 (1.2)	43 (6.7)	57 (8.6)

Data are expressed as an unweighted number (%).

*Vitamin D deficiency was defined as blood 25(OH)D3 <20 ng/mL. Vitamin D insufficiency was defined as a blood 25(OH)D3 concentration of 20–30 ng/mL.

†Adequate intake of vitamin D for younger and middle-aged adults is 10 μg/day and that for older adults is 15 μg/day.

‡Low muscle mass was defined as appendicular skeletal muscle index (Appendicular skeletal muscle mass/height^2) <7.0 kg/m^2 for men and <5.7 kg/m^2 for women.

§Low muscle strength was defined as maximal handgrip strength <28 kg for men and <18 kg for women.

‖Sarcopenia was defined as low muscle mass plus low muscle strength.

Table S2. General characteristics of participants aged 19–39 years by vitamin D status

Characteristics	Younger men (n = 447)		P-value*	Younger women (n = 549)		P-value*
	Normal (n = 171)	Vit D deficiency (n = 276)		Normal (n = 223)	Vit D deficiency (n = 326)	
Age, years	30.9 ± 0.5	28.5 ± 0.3	<0.001	31.4 ± 0.4	27.6 ± 0.4	<0.001
Household income			0.353			0.082
Low	17 (11.4)	33 (12.8)		16 (7.5)	39 (12.6)	
Middle-low	26 (13.4)	56 (19.3)		48 (19.9)	93 (26.5)	
Middle-high	62 (35.9)	95 (34.9)		87 (35.5)	110 (32.6)	
High	66 (39.3)	92 (33.0)		72 (37.1)	84 (28.3)	
Education			0.676			<0.001
Low	0 (0)	1 (0.4)		1 (0.3)	1 (0.4)	
Middle-low	1 (1.1)	3 (0.9)		0 (0.0)	4 (1.6)	
Middle-high	69 (39.3)	128 (44.2)		49 (18.7)	130 (40.5)	
High	101 (59.7)	144 (54.5)		173 (80.9)	191 (57.4)	
Current alcohol consumer	114 (64.4)	180 (64.8)	0.933	127 (55.6)	197 (59.3)	0.434
Current smoker	47 (26.6)	80 (29.1)	0.641	7 (2.6)	25 (8.0)	0.014
Regular resistance exercise	81 (48.9)	86 (31.4)	0.001	52 (23.2)	67 (21.8)	0.716
Body mass index, kg/m^2	25.1 ± 0.3	25.3 ± 0.3	0.522	22.1 ± 0.2	22.7 ± 0.3	0.069
Appendicular skeletal muscle index†	8.13 ± 0.06	8.02 ± 0.05	0.148	6.13 ± 0.04	6.15 ± 0.05	0.846
Maximal handgrip strength	44.6 ± 0.5	42.2 ± 0.6	0.003	25.8 ± 0.4	25.7 ± 0.3	0.792
Low muscle mass‡	14 (7.5)	27 (10.0)	0.384	58 (25.3)	89 (28.1)	0.516
Low muscle strength§	2 (1.0)	10 (3.6)	0.069	12 (4.4)	12 (3.5)	0.609
Sarcopenia‖	1 (0.5)	4 (1.6)	0.189	9 (3.1)	7 (2.0)	0.684
Blood 25(OH)D3 level, ng/mL	27.2 ± 0.6	13.1 ± 0.3	<0.001	27.6 ± 0.6	13.2 ± 0.2	<0.001
Total energy intake, kcal/d	2186 ± 60	2095 ± 47	0.222	1527 ± 38	1607 ± 40	0.138
Energy from protein, %	16.4 ± 0.4	16.0 ± 0.3	0.413	16.6 ± 0.4	15.7 ± 0.3	0.040
Vitamin D intake, µg/d	3.2 ± 0.5	2.9 ± 0.2	0.608	2.6 ± 0.3	2.6 ± 0.3	0.953
Adequate vitamin D intake¶	9 (5.5)	13 (5.0)	0.856	5 (3.1)	13 (4.0)	0.664

Data are expressed as means ± standard error for continuous variables or numbers (%) for categorical variables.

*Differences between consumers and non-consumers were determined by t-test for continuous variables and Rao-Scott chi-square test for categorical variables.

†Appendicular skeletal muscle index was calculated by dividing the sum of appendicular muscle mass by the square of the height.

‡Low muscle mass was defined as appendicular skeletal muscle index (Appendicular skeletal muscle mass/height^2) <7.0 kg/m^2 for men and <5.7 kg/m^2 for women.

§Low muscle strength was defined as maximal handgrip strength <28 kg for men and <18 kg for women.

‖Sarcopenia was defined as low muscle mass plus low muscle strength.

¶Adequate intake of vitamin D for younger adults is 10 µg/day.

Table S3. General characteristics of participants aged 40–64 years by vitamin D status

Characteristics	Middle-aged men (n = 731)			Middle-aged women (n = 1057)		
	**Normal (n = 409)**	**Vit D deficiency (n = 322)**	**P-value***	**Normal (n = 667)**	**Vit D deficiency (n = 390)**	**P-value***
Age, years	51.8 ± 0.4	50.9 ± 0.4	0.086	53.4 ± 0.3	50.2 ± 0.4	<0.001
Household income			0.265			0.214
Low	34 (6.4)	26 (7.0)		74 (9.3)	30 (7.7)	
Middle-low	74 (16.8)	57 (16.9)		146 (20.2)	102 (24.3)	
Middle-high	117 (29.7)	110 (36.0)		212 (35.5)	123 (29.9)	
High	184 (47.1)	129 (40.1)		235 (35.0)	135 (38.0)	
Education			0.149			0.019
Low	23 (4.0)	8 (1.6)		58 (6.9)	17 (4.1)	
Middle-low	24 (4.4)	24 (6.8)		54 (7.1)	24 (4.7)	
Middle-high	140 (33.7)	113 (35.0)		285 (46.3)	154 (41.8)	
High	219 (57.9)	175 (56.6)		259 (39.8)	193 (49.4)	
Current alcohol consumer	292 (72.2)	238 (73.7)	0.678	281 (40.5)	173 (45.0)	0.248
Current smoker	130 (29.9)	133 (40.6)	0.007	29 (4.2)	21 (4.2)	0.970
Regular resistance exercise	117 (28.6)	87 (26.7)	0.606	113 (16.2)	45 (10.8)	0.058
Body mass index, kg/m^2	25.0 ± 0.2	25.4 ± 0.2	0.178	23.3 ± 0.2	23.8 ± 0.2	0.044
Appendicular skeletal muscle index†	8.04 ± 0.04	8.02 ± 0.04	0.647	6.25 ± 0.03	6.33 ± 0.04	0.095
Maximal handgrip strength	43.0 ± 0.4	42.5 ± 0.4	0.380	25.4 ± 0.2	25.7 ± 0.2	0.381
Low muscle mass‡	31 (5.9)	20 (5.0)	0.618	114 (17.9)	51 (14.4)	0.232
Low muscle strength§	6 (0.8)	4 (1.4)	0.405	23 (2.9)	18 (3.7)	0.530
Sarcopenia‖	2 (0.3)	1 (0.4)	0.637	11 (1.4)	5 (0.8)	0.252
Blood 25(OH)D3 level, ng/mL	29.1 ± 0.4	14.0 ± 0.2	<0.001	31.5 ± 0.5	13.7 ± 0.2	<0.001
Total energy intake, kcal/d	2075 ± 33	2073 ± 39	0.973	1533 ± 21	1579 ± 31	0.247
Energy from protein, %	15.4 ± 0.2	15.1 ± 0.2	0.255	15.6 ± 0.2	15.4 ± 0.2	0.434
Vitamin D intake, µg/d	4.0 ± 0.3	3.2 ± 0.2	0.061	2.9 ± 0.2	2.6 ± 0.2	0.479
Adequate vitamin D intake¶	31 (8.3)	21 (6.1)	0.336	22 (3.0)	14 (3.6)	0.639

Data are expressed as means ± standard error for continuous variables or numbers (%) for categorical variables.

*Differences between consumers and non-consumers were determined using the t-test for continuous variables and Rao-Scott chi-square test for categorical variables.

†Appendicular skeletal muscle index was calculated by dividing the sum of appendicular muscle mass by the square of the height.

‡Low muscle mass was defined as appendicular skeletal muscle index (Appendicular skeletal muscle mass/height^2) <7.0 kg/m^2 for men and <5.7 kg/m^2 for women.

§Low muscle strength was defined as maximal handgrip strength <28 kg for men and <18 kg for women.

‖Sarcopenia was defined as low muscle mass plus low muscle strength.

¶Adequate intake of vitamin D for middle-aged adults is 10 µg/day.

Table S4. General characteristics of participants aged ≥65 years by vitamin D status

Characteristics	Older men (n = 547)		P-value*	Older women (n = 589)		P-value*
	Normal (n = 361)	Vit D deficiency (n = 196)		Normal (n = 443)	Vit D deficiency (n = 168)	
Age, years	72.0 ± 0.3	72.9 ± 0.5	0.096	72.0 ± 0.3	72.3 ± 0.5	0.629
Household income			0.145			0.375
Low	123 (31.5)	80 (37.7)		192 (41.7)	83 (49.4)	
Middle-low	133 (40.0)	57 (30.3)		137 (31.7)	39 (25.3)	
Middle-high	56 (16.2)	36 (21.2)		60 (15.1)	24 (15.9)	
High	43 (12.4)	19 (10.8)		39 (11.4)	13 (9.5)	
Education			0.282			0.575
Low	101 (27.0)	64 (33.4)		240 (55.5)	85 (56.6)	
Middle-low	72 (21.7)	21 (13.5)		62 (15.9)	20 (13.8)	
Middle-high	94 (28.8)	53 (27.6)		72 (18.5)	30 (22.9)	
High	66 (22.5)	38 (25.5)		35 (10.1)	9 (6.7)	
Current alcohol consumer	209 (59.9)	97 (52.2)	0.100	75 (16.4)	27 (19.2)	0.454
Current smoker	75 (18.7)	35 (18.4)	0.942	8 (1.7)	1 (0.5)	0.254
Regular resistance exercise	105 (31.9)	60 (34.6)	0.519	67 (16.3)	20 (11.3)	0.168
Body mass index, kg/m^2	23.9 ± 0.2	23.9 ± 0.2	0.934	24.0 ± 0.2	25.0 ± 0.3	0.015
Appendicular skeletal muscle index†	7.44 ± 0.04	7.32 ± 0.06	0.062	6.00 ± 0.03	6.14 ± 0.06	0.062
Maximal handgrip strength	35.8 ± 0.3	34.1 ± 0.5	0.006	22.5 ± 0.2	22.2 ± 0.4	0.586
Low muscle mass‡	89 (24.0)	70 (32.9)	0.024	134 (30.6)	38 (22.5)	0.073
Low muscle strength§	39 (9.7)	38 (18.1)	0.013	61 (12.2)	29 (17.6)	0.126
Sarcopenia‖	19 (4.4)	24 (10.2)	0.019	42 (8.6)	15 (8.8)	0.086
Blood 25(OH)D3 level, ng/mL	30.1 ± 0.4	14.3 ± 0.4	<0.001	34.4 ± 0.6	14.4 ± 0.3	<0.001
Total energy intake, kcal/d	1884 ± 35	1900 ± 59	0.814	1443 ± 30	1452 ± 46	0.869
Energy from protein, %	14.8 ± 0.2	14.3 ± 0.3	0.219	14.7 ± 0.2	14.1 ± 0.3	0.097
Vitamin D intake, µg/d	3.5 ± 0.3	2.8 ± 0.3	0.135	2.4 ± 0.2	2.2 ± 0.4	0.647
Adequate vitamin D intake¶	8 (2.1)	3 (1.2)	0.415	5 (1.2)	2 (2.0)	0.593

Data are expressed as means ± standard error for continuous variables or numbers (%) for categorical variables.

*Differences between consumers and non-consumers were determined by t-test for continuous variables and Rao-Scott chi-square test for categorical variables.

†Appendicular skeletal muscle index was calculated by dividing the sum of appendicular muscle mass by the square of the height.

‡Low muscle mass was defined as appendicular skeletal muscle index (Appendicular skeletal muscle mass/height^2) <7.0 kg/m^2 for men and <5.7 kg/m^2 for women.

§Low muscle strength was defined as maximal handgrip strength <28 kg for men and <18 kg for women.

‖Sarcopenia was defined as low muscle mass plus low muscle strength.

¶Adequate intake of vitamin D for older adults is 15 µg/day.

Table S5. Association between vitamin D status and the risk of low muscle mass, low muscle strength, and sarcopenia among older adults

Blood 25(OH)D3 level	Unadjusted		Model 1*		Model 2†		Model 3‡	
	OR	95% CI	OR	95% CI	OR	95% CI	OR	95% CI
Low muscle mass								
Men								
Normal	1 (ref)		1 (ref)		1 (ref)		1 (ref)	
Vitamin D insufficiency	0.65	0.38, 1.13	0.64	0.32, 1.27	0.68	0.34, 1.36	0.68	0.34, 1.37
Vitamin D deficiency	1.21	0.73, 2.02	1.37	0.76, 2.48	1.44	0.78, 2.66	1.46	0.80, 2.65
Women								
Normal	1 (ref)		1 (ref)		1 (ref)		1 (ref)	
Vitamin D insufficiency	0.72	0.48, 1.09	1.04	0.60, 1.81	1.03	0.56, 1.90	1.01	0.55, 1.84
Vitamin D deficiency	0.58	0.36, 0.95	0.82	0.44, 1.51	0.73	0.39, 1.34	0.72	0.37, 1.34
Low muscle strength								
Men								
Normal	1 (ref)		1 (ref)		1 (ref)		1 (ref)	
Vitamin D insufficiency	0.85	0.44, 1.64	0.91	0.46, 1.78	0.92	0.48, 1.77	0.92	0.48, 1.76
Vitamin D deficiency	1.87	0.97, 3.58	1.79	0.92, 3.48	1.76	0.92, 3.36	1.71	0.91, 3.23
Women								
Normal	1 (ref)		1 (ref)		1 (ref)		1 (ref)	
Vitamin D insufficiency	0.97	0.51, 1.83	1.04	0.52, 2.08	1.05	0.53, 2.05	1.07	0.56, 2.08
Vitamin D deficiency	1.51	0.82, 2.78	1.64	0.86, 3.14	1.42	0.72, 2.79	1.46	0.75, 2.84
Sarcopenia								
Men								
Normal	1 (ref)		1 (ref)		1 (ref)		1 (ref)	
Vitamin D insufficiency	1.83	0.69, 4.85	1.95	0.73, 5.24	1.96	0.73, 5.26	1.97	0.74, 5.29
Vitamin D deficiency	3.83	1.45, 10.13	3.99	1.46, 10.93	3.65	1.30, 10.25	3.59	1.28, 10.07
Women								
Normal	1 (ref)		1 (ref)		1 (ref)		1 (ref)	
Vitamin D insufficiency	0.65	0.31, 1.33	0.77	0.33, 1.79	0.75	0.32, 1.75	0.77	0.33, 1.79
Vitamin D deficiency	0.94	0.45, 1.98	1.11	0.47, 2.63	0.84	0.35, 2.04	0.87	0.36, 2.09

Vitamin D deficiency was defined as blood 25(OH)D3 <20 ng/mL. Vitamin D insufficiency was defined as a blood 25(OH)D3 concentration of 20–30 ng/mL. Low muscle mass was defined as appendicular skeletal muscle index (Appendicular skeletal muscle mass/height^2) <7.0 kg/m^2 for men and <5.7 kg/m^2 for women.

*Model 1: adjusted for age, body mass index, and total energy intake

†Model 2: adjusted for all covariates included in model 1 plus household income, alcohol consumption, smoking, and resistance exercise

‡Model 3: adjusted for all covariates included in model 2 plus energy from protein
